# United Against Rabies Forum: The first 2 years

**DOI:** 10.3389/fpubh.2023.1010071

**Published:** 2023-03-23

**Authors:** Rachel Tidman, Anna Sophie Fahrion, S. M. Thumbi, Ryan M. Wallace, Katinka De Balogh, Vivian Iwar, Gowri Yale, Isabelle Dieuzy-Labaye

**Affiliations:** ^1^World Organisation for Animal Health, Paris, France; ^2^Institute of International Animal Health/One Health, Friedrich-Loeffler-Institute, Greifswald-Riems, Germany; ^3^Center for Epidemiological Modelling and Analysis, University of Nairobi, Nairobi, Kenya; ^4^Institute of Immunology and Infection Research, University of Edinburgh, Edinburgh, United Kingdom; ^5^Paul G. Allen School for Global Health, Washington State University, Pullman, WA, United States; ^6^Centers for Disease Control and Prevention, Atlanta, GA, United States; ^7^Food and Agriculture Organization of the United Nations, Rome, Italy; ^8^Economic Community of West African States Commission, Abuja, Nigeria; ^9^Mission Rabies, Panjim, India

**Keywords:** rabies, United Against Rabies Forum, rabies elimination, Zero by 30, One Health, neglected tropical diseases, zoonosis

## Abstract

Rabies continues to kill an estimated 59,000 people annually, with up to 99% of human cases transmitted by domestic dogs. The elimination of human deaths from dog-mediated rabies is achievable by applying a One Health approach, and the framework to do this is outlined in Zero by 30: the Global Strategic Plan to end human deaths from dog-mediated rabies by 2030. To build on this global goal, and implement the approaches set out in Zero by 30, the United Against Rabies Forum was launched in 2020. This paper gives a review of the objectives, governance, activities and achievements of the United Against Rabies Forum to date. It also outlines ongoing work, and next steps as the United Against Rabies Forum reviews its first 2 years of activities and identifies priority areas for the coming 12 months.

## Introduction

Rabies, one of the world's most ancient infectious diseases and most lethal viral zoonosis of mammals, kills an estimated 59,000 people annually across large regions of the globe (1). Transmitted by domestic dogs in up to 99% of human cases, effective control methods to tackle the disease have been long proven but often do not reach the population in need—poor and marginalized communities (2). The elimination of human deaths from dog-mediated rabies is achievable by applying a One Health approach, and the framework to do this is outlined in Zero by 30: the Global Strategic Plan to end human deaths from dog-mediated rabies by 2030 (*Zero by 30*) (3). Developed by the Food and Agriculture Organisation of the United Nations (FAO), World Organisation for Animal Health (WOAH), World Health Organisation (WHO) (the Tripartite) and the Global Alliance for Rabies Control (GARC) in 2018, this country-centric strategy prioritizes the changes needed to reach zero human deaths from dog-mediated rabies with three core objectives: to effectively use vaccines, medicines, tools and technologies; to generate, innovate, and measure impact; and to sustain commitment and resources (3).

To use the momentum created by *Zero by 30*, the Tripartite launched the United Against Rabies Forum (UAR Forum) in 2020 to create a broad and inclusive network of stakeholders who share a vision for the elimination of dog-mediated rabies, and wish to work collectively to achieve it (4). Three working groups, encompassing more than 30 institutions, were established to progress activities that contributed to each of the three *Zero by 30* objectives (4). In line with a One Health approach, these working groups actively engage a wide range of stakeholders including participants from national and regional entities, human and animal health sectors, NGOs, academic and research institutions, and private sectors (4). This paper gives a review of the objectives, governance, activities and achievements of the UAR Forum to date. It also outlines ongoing work, and next steps as the UAR Forum reviews the first 2 years of activities and identifies priority areas for the coming 12 months.

## UAR Forum governance and mode of operation

*Zero by 30* established an achievable goal and a common plan for the international community to work toward. However, further work was needed to actively bring stakeholders together. To address this, the Tripartite set out to establish a network, the UAR Forum, that could bring partners together to collectively implement the objectives set out in the global strategy, and accelerate progress toward their shared vision of ending human deaths from dog-mediated rabies.

The UAR Forum is hosted by WOAH, on behalf of the Tripartite organizations, with the Terms of Reference undergoing review by focal points and legal teams of each organization until these were agreed upon. The Tripartite maintains oversight of the UAR Forum, with strategic direction provided by the Steering Group, which is comprised of representatives of the three Tripartite organizations and chairs of each working group (5). The Steering Group meets quarterly to review progress, ensure that working group activities are coordinated and synergistic, and provide strategic direction in planning next steps.

The wider UAR Forum consists of member organizations that are committed to implementing the approaches set out in *Zero by 30*, while sharing knowledge, experience, ideas and information. UAR Forum members include intergovernmental and non-governmental organizations, national governmental institutions, private sector entities, philanthropic foundations and academic and research institutions. At the time of publication, more than 50 organizations have become members of the UAR Forum. Organizations can view the UAR Forum Terms of Reference and apply for membership through the UAR Forum website (www.UnitedAgainstRabies.org).

To ensure that practical outputs were developed to support countries in progressing with control and elimination of dog-mediated rabies, results-focused working groups were established to address priority activities that contributed to the three objectives outlined in *Zero by 30*. Three working groups were established in 2021: Working Group 1—Effective use of vaccines, medicines, tools and technologies; Working Group 2—Strategic and operational support to countries; Working Group 3—Advocacy and resource mobilization. The workplans of each working group initially focused on priority activities that were realistic and could be developed over a 12–18 month period, with the intention of expanding the workplan to include further activities at a later date.

Participation in the working groups is not mandatory for UAR members. Instead working groups consist of participants that represent a small a number of UAR member organizations, and have expressed their interest and availability in actively contributing to focused activities (5). Working group chairs provide coordination and leadership for working group activities and lead working group discussions to prioritize topics and review progress. Workstreams are developed within each working group, with a workstream lead nominated to coordinate the work. These workstreams meet virtually on a regular basis to progress activities, reporting back to the larger working group every 2–4 weeks. To ensure the working groups remain inclusive and productive, participation is flexible, with workstream participants contributing to multiple workstreams over time, or providing short-term contributions to specific activities. Contribution in the working groups is unpaid and voluntary, with each working group participant dedicating time and expertise according to their individual availability and capacity. Stakeholders interested in finding out more or contributing to specific activities can contact the UAR Forum through the contact page of www.UnitedAgainstRabies.org.

## Key achievements

### Facilitating knowledge transfer—The United Against Rabies Forum webinars and website

The inaugural stakeholder event, entitled “United Against Rabies: One Health in Action—Partnering for Success” was held virtually in September 2020 (6). This event not only acted to officially launch the UAR Forum, but provided an opportunity for stakeholders to define and recommend the priority areas that should be progressed by UAR Forum working groups. In 2021, a series of three webinars were held in September and October to update stakeholders on the progress and outputs of the UAR Forum, and post-webinar evaluation forms were also disseminated to allow stakeholders to provide feedback on the priority areas that should be addressed, and areas that needed to be reviewed (7). Between 200 and 400 participants representing both human and animal health sectors from 92 countries attended each webinar (7). A further webinar was held in 2022, focusing this time on a single priority area that stakeholders had identified—“Tackling Rabies and Dog Population Management: the Role of Local Authorities” (8).

UAR Forum members also presented on the UAR Forum activities and outputs in more than 20 virtual and in-person events over the course of 2021 and 2022, advocating for *Zero by 30*, and inviting stakeholders to contribute to the collective effort. However, as much of the engagement was virtual over the first 12 months of the UAR Forum due to the COVID-19 pandemic, and there was no central platform to direct stakeholders to, there were challenges in achieving an inclusive network and disseminating Forum outputs. The UAR Forum website (www.UnitedAgainstRabies.org) was launched in early 2022 and helped overcome these challenges by providing a central platform where stakeholders could access technical resources, sign up for news and events and express interest in UAR Forum membership.

To improve engagement and communication of updates and UAR Forum outputs with stakeholders, the UAR Forum will hold webinars on a quarterly basis, focusing on key priority areas or newly developed resources that can support stakeholders in implementing rabies control programmes. Webinars will be open to all, and provide an opportunity for participants to network and ask questions of experts.

Quarterly newsletters will also be disseminated to the UAR Forum mailing list, providing news, updates on upcoming events, latest outputs and inviting stakeholders to participate in UAR Forum surveys where relevant. Individuals can sign up to the UAR Forum mailing list at www.unitedagainstrabies.org, or contact the United Against Rabies Forum by emailing globalrabiescoordinator@woah.org.

### Working group workstreams

Within each of the three working groups, several workstreams were established to focus on specific priority topics. A working group member was identified as the workstream lead, and were responsible for leading the workstream discussions and sharing progress and results with the wider working group on a regular basis. Working group outputs were made available for review by all working groups, before being submitted to the Steering Group for final review. Following approval by the Steering Group, working group outputs were finalized and made publicly available. Several of the completed workstreams, and the respective outputs from these, are discussed below, with Table 1 providing direct links to key outputs of the UAR Forum to date.

**Table 1:** Links to key outputs of the UAR Forum.

**Key outputs**	**Link**
United Against Rabies website	www.UnitedAgainstRabies.org
Webinars	2020: One Health in Action: Partnering for Success
	2021 (series of three webinars): Rabies, One Health and Covid 19; Data gaps, monitoring and tools and technology; National Rabies Control Programmes
	2022: Tackling Rabies and Dog Population Management: the Role of Local Authorities
Template for national strategic plan to control rabies	Access template in English and French
Minimum data elements for surveillance	Access document in English
United Against Rabies toolbox	Visit Toolbox
United Against Rabies roadmap	Visit Roadmap
One Health Joint Plan of action—rabies embedded within “action track 3: neglected zoonotic diseases”	Read the One Health Joint Plan of Action here
Template for landscaping of resource partners for rabies	Access template in English
Case studies	Rabies prevention and control—lessons from Chonqing, China
	How Mexico achieved rabies-free status
	Goa State and Mission Rabies—achieving rabies-free status together
	Namibia's national plan for rabies elimination delivers benefits
Example pitch deck	Access example pitch deck

Experts within the Forum are available to provide stakeholders with additional information or support to better utilize and implement any of the UAR Forum outputs, and contact can be made through the UAR Forum website to request support, or express interest in contributing to specific areas of work.

#### National strategic plan template

A critical aspect of *Zero by 30* is that it focuses on country-centric engagement, with countries leading efforts. A key milestone for countries to do this is the development of a national strategic plan—providing a framework outlining targets and measurable deliverables, and demonstrating government commitment to the global goal of *Zero by 30*. However, developing this strategy can often be a challenge for countries. To address this, participants of Working Group 2 (Strategic and operational support) reviewed available documents and existing resources (3, 9–18) to develop a national strategic plan template that follows international guidelines and standards, to help countries develop their own robust, One Health focused canine rabies elimination plan. The template is flexible enough to provide practical guidance for countries in early stages of canine rabies elimination, and countries at more advanced stages, and aligns with the WOAH process for the endorsement of official control programmes for dog-mediated rabies (9). Countries that achieve this endorsement will likely increase chances of obtaining national and international funding (9). The template has been disseminated through regional networks, and used to support the development of several national strategic plans to date, including Burkina Faso, Cambodia, Sierra Leone and Togo. The template will be reviewed and updated regularly according to feedback from national stakeholders, to ensure that this remains useful and fit for purpose. It is available in English and French on the UAR Forum website, and will be linked to the UAR Forum roadmap (discussed below) as a resource to help countries in achieving milestones associated with the development and implementation of a national strategy (19).

#### Surveillance and minimum data elements

Surveillance is a critical component of national control programmes. Despite rabies being one of the deadliest diseases known to man, in practice it remains a hidden disease due to poor surveillance and underreporting. Rabies-related data span the One Health spectrum, with human deaths and human vaccination data collected by human health authorities (most often Ministries of Health), whereas animal rabies cases and animal vaccination data are often collected by Veterinary Authorities (often Ministries of Agriculture). This has led to constraints in data sharing, and the development of numerous and overlapping guidance documents and data definitions.

Data should be collected for a specific purpose, be that informing interventions (e.g., Post-Exposure Prophylaxis (PEP) or dog vaccination programmes), monitoring trends, or evaluating the effectiveness of disease programmes. Consistent case definitions are a foundational requirement for surveillance programmes, as well as monitoring and evaluation of progress toward *Zero by 30*, and data elements are often informed by standard case definitions. Participants in Working Group 1 (Effective use of vaccines, medicines, tools and technologies) set out to examine commonly used case definitions for rabies programmes and identify the minimum data elements that inform the current status and progress of National rabies control programmes. A landscape analysis of available documents was conducted (2, 9, 20–29), and numerous inconsistencies were identified in national, regional and international data element definitions commonly used by rabies programmes.

To address this, Working Group 1 developed a comprehensive document that provides realistic set of essential data elements that are needed to track progress toward *Zero by 30*, allowing for reliable, comparable estimates in time and between countries (30). Furthermore, the document provides consistent definitions for key case definitions and data elements that align with global standards published by WHO and WOAH (2, 22). Standardizing terminology is essential for communication and comprehension of information between international advisory forums, regional implementation partners and national administrators. National authorities are encouraged to use the minimum data elements guide to adopt globally standardized data definitions, improve in-country rabies data collection and submit data to the WHO Global Health Observatory and the WOAH World Animal Health Information System on an annual basis (24, 25). By providing a common standard for rabies data, the aim is to move toward resolving rabies-related information gaps that would improve health policy decisions globally.

This document has been disseminated through regional networks, and will be a key topic in an upcoming UAR webinar in 2023, entitled “Rabies surveillance: what gets measured gets done.” The UAR Forum includes members that are experts in rabies surveillance, and stakeholders can contact the UAR Forum if support is needed in adopting these standardized data elements.

#### Tool evaluation

Despite a number of effective tools and applications being available to support national control programmes and canine rabies elimination efforts (e.g., tools for economic modeling, planning and implementation of dog vaccination and IBCM, monitoring and evaluation of national rabies programmes), these are often inaccessible or underutilized by stakeholders. Working Group 1 participants focused on collecting existing tools for canine rabies elimination, evaluating these, and providing guidance to countries on how to adapt and use these to improve their rabies programmes. The identified tools and technical resources have been aligned to the workplan of the Stepwise Approach for Rabies Elimination (SARE) (11), to help stakeholders identify tools that are appropriate and fit for purpose for specific activities. The UAR Forum website provides an interface where users can identify the category of tool they need, the strengths and weaknesses of the tool, then contact developers if required to integrate this into their programme (31).

#### Roadmap

Although many countries have made progress toward freedom from dog-mediated rabies, countries are at differing stages, and often find it challenging to determine the steps and approaches that should be implemented to scale up efforts, and tools and resources that can be utilized to progress further. The development of the UAR Forum roadmap aims to help countries monitor their progress toward canine rabies elimination using a standardized approach with clear progress milestones, while also introducing countries to the most appropriate tools and resources for their activities. In providing an objective roadmap score for a country, aligned with international criteria and milestones of WHO and WOAH (2), this roadmap will also help monitor progress at a global level. National stakeholders can establish the roadmap score for their country by completing a SARE assessment (11). The SARE assessment helps users to assess the strengths and weaknesses of a national rabies control programme, assists the user with developing a rabies workplan, and provides a SARE score indicating the current rabies situation across a country—a score which provides the basis for the roadmap score. The roadmap builds upon this, by bringing together specific tools and resources, including the aforementioned working group outputs, that are readily available to address pending activities in the country's canine rabies elimination work plan, and thereby provide tangible steps for countries to take to progress to the next milestone. For example, national stakeholders at an early stage of the roadmap with no national strategic plan will be directed to the national strategic plan template, stakeholders identifying a need to improve surveillance will be directed to the minimum data elements document, and specific activities identified within a country workplan will link to tools in the UAR toolbox that can help in implementing these activities. The roadmap is available on the UAR Forum website, and further work on this is anticipated in 2023 to provide direct links to tools and resources (32).

#### Integration of rabies into the One Health Joint Plan of Action

The COVID-19 pandemic highlighted the need for a One Health approach in overcoming challenges and emerging threats associated with zoonotic diseases. In response to a global call for action, the Food and Agriculture Organisation of the United Nations (FAO), the UN Environment Programme (UNEP), the World Health Organisation (WHO), and the World Organisation for Animal Health (WOAH) (the Quadripartite) launched the One Health Joint Plan of Action (OH JPA) in October 2022 (33). This five-year plan aims to create a framework of actions that contribute to One Health, while aligning and building on existing One Health and coordination initiatives, including *Zero by 30* (33).

The Tripartite and members of the UAR Forum worked to ensure the inclusion of rabies in this wider One Health agenda, resulting in the integration of rabies in Action Track 3 of the OH JPA, “Controlling and eliminating endemic zoonotic, neglected tropical and vector-borne diseases” (33). Having rabies embedded into the OH JPA will provide support for advocacy efforts, demonstrating to investors that their investment is contributing to a plan that is widely and most visibly endorsed by the international community.

#### Landscaping of national resource partners

Mobilizing domestic and international resources is critical for effectively and sustainably implementing national control programmes. For planning purposes, it is key to identify agencies/organizations that have funded rabies prevention programmes/initiatives in the past, and also to identify resource partners that can provide required resources (including non-financial resources e.g., equipment, consumables, infrastructure, IT or communication services). To assist national stakeholders, Working Group 3 has developed a template to help map and profile potential resource partners that can support the development and implementation of their national control programme. This template can be accessed on the UAR Forum website (34).

#### Case studies

Four case studies have been developed, and are available on the UAR Forum website (Mexico; Namibia; Goa, India; Chongqing, China) (35). Each case study demonstrates unique strategies and specific partnerships, but these all highlight the catalytic role that resource partners have played in canine rabies elimination. The focus of Working Group 3 was to adapt these case studies in a way that could target investors, advocating for their engagement toward canine rabies elimination, and showing how this could be replicated in other areas with the right support. These case studies have been instrumental in discussions with international investment partners, helping to demonstrate the impact that their investment could have in progressing toward canine rabies elimination.

#### Pitch deck

Working Group 3 participants worked to develop resources that could support stakeholders in developing their own canine rabies elimination advocacy and investment pitches. Participants reviewed and collated a vast amount of rabies related material, before consolidating this and working with communication experts to transform targeted information into an example pitch deck template to support investment in canine rabies elimination. Stakeholders are able to download an example pdf slide deck, and use this information to develop their own pitch to potential investors. The pitch deck is supported by instructions for helping stakeholders develop the best messaging and targeting for specific categories of investors (36).

#### Identification of main constraints to rabies control

The objective of this workstream was to identify, analyse and provide information on constraints preventing countries from progressing toward *Zero by 30* and to link to potential solutions. To provide an evidence base, participants carried out a scoping review of the literature, extracting and compiling mentioned constraints and clustering these into overarching categories. These constraint categories were used as a basis for virtual polls (conducted during the 2021 UAR webinar series, 2021 virtual Rabies in the Americas conference, and a 2022 workshop conducted in Côte d'Ivoire), with participants asked to prioritize constraints based on their experiences. This prioritization informed the establishment of a new workstream in Working Group 1, focused on improving dog vaccination coverage (one of the prioritized constraints by poll participants). Investigating such priorities in more depth could help to identify patterns for the most pressing constraints depending on factors such as geography or level of canine rabies elimination progress already achieved, directing support and resources into more focused and meaningful directions.

## Ongoing workstreams

### Linking tools, the roadmap and the canine rabies blueprint

Further work is underway to integrate the outputs of the tool evaluation and roadmap workstreams, specifically looking at linking specific tools and resources within the roadmap—in this way countries can identify both the key activities they need to undertake to progress to the next roadmap milestone, and the specific tools that can help them achieve these activities. The tool evaluation workstream will also progress with reaching out to tool developers, encouraging them to submit their tools for evaluation by experts, so that these can be included in the UAR Forum toolbox. Tool developers who wish to submit their tools for evaluation can contact the UAR Forum *via* the UAR Forum website.

The canine rabies blueprint is also an online repository of resources developed by a global panel of rabies experts, however the website and much of the content is outdated and in need of review and updating (12). Participants of the UAR Forum working groups aim to review the existing resources and content on the canine rabies blueprint in 2023, update or develop new content where required, and integrate this with the UAR Forum website and toolbox to ensure its ongoing availability and use of the relevant resources.

### Partnership map

Despite the increased global commitment toward the elimination of human deaths from dog-mediated rabies, rabies control efforts remain fragmented and uncoordinated in many areas, resulting in duplicated efforts, poor use of resources and ineffective or unsustainable activities. To address these challenges and to improve coordination, transparency and equitable support to canine rabies endemic countries, the UAR Forum is developing a partnership map. This map will provide a regional and global overview of rabies stakeholders and will foster collaboration between stakeholders, helping them to better align efforts, avoid duplication and identify synergies. This workstream is currently collecting data on UAR Forum member activities (types of activity, and where these are being conducted), after which this will inform the development of a publicly available map which will be displayed on the UAR Forum website.

### Improving the human-animal bond with dog identification

Despite decades of evidence that dog vaccination is the most cost-effective means to reduce dog-mediated human rabies deaths, most canine rabies endemic countries do not reach needed vaccination threshold coverages to eliminate the virus. Anecdotal evidence from workstream members has suggested that ways to identify vaccinated dogs (e.g., through a collar provided at the time of vaccination) can improve participation in dog vaccination campaigns and have other downstream positive impacts. However, dog identification comprises an additional expense for under-funded vaccination programmes. The objective of this workstream is to use ideas from theory of change literature to develop pilot projects and to collect data to demonstrate that when people can differentiate between vaccinated and unvaccinated dogs, there will be a change in behavior between people and dogs, as well as more responsible dog ownership through community pressure on dog owners. It is hypothesized that this change in human-dog interactions can lead to reductions in dog bites, and therefore reductions in the need for post-exposure prophylaxis. Additionally, improved human-dog bonds may decrease culling of stray dogs and improve utilization of routine veterinary services. These benefits have tangible and intangible benefits that may justify the additional expense of dog identification. The workstream are planning to implement pilot projects in a number of countries in order to collect robust data to help demonstrate the cost-effectiveness of combined dog vaccination and dog identification.

### Dog vaccination

Mass dog vaccination interrupts the rabies transmission cycle and is a critical pillar of canine rabies elimination strategies. However, the constraints workstream of Working Group 2 identified improving mass dog vaccination coverage as a main priority for stakeholders. While the improving human-animal bond with dog identification workstream is focused primarily on how dog identification can improve dog vaccination, this workstream is focusing on conducting further stakeholder analysis to determine what constraints or barriers are experienced in planning, implementing or evaluating dog vaccination campaigns, in order to identify practical solutions in overcoming these constraints.

### Rapid diagnostic testing

Rapid diagnostic tests (RDTs) are diagnostic assays designed for use at the point-of-care, and can be adapted for use in low-resource settings (37, 38). Accurate, sensitive and affordable diagnostic methods could be a game changer for canine rabies elimination, and RDTs and home-based testing have proven pivotal tools in achieving reductions in other major infectious diseases being targeted for elimination. RDTs for rabies have been previously evaluated by the WOAH Reference Laboratories, and found to have low sensitivity and specificity, as well as inconsistent performance between products and batches. However, recent field-based evidence is showing promising results using a modified protocol (39–42). This workstream aims to review evidence on RDT performance for rabies diagnosis, analyse costs and benefits of diagnostic test modalities, investigate potential for RDT certification, develop guidance to support policy and practice on RDTs and investigate funding opportunities to support wider RDT use.

### Oral rabies vaccine recommendations

Oral rabies vaccination (ORV) is conducted in numerous countries for the control of wildlife rabies, but has not been used in any meaningful capacity to control dog-mediated rabies. One constraint to use of ORV in dogs is the lack of international guidance for the selection of safe and effective products and how to incorporate ORV into a large-scale dog vaccination programmes. Guidance for ORV in dogs was last updated in 2007 (43), and does not reflect major advances in the production, safety, efficacy and recent field-use data for ORV in dogs. This workstream is revising the 2007 WHO Recommendations on ORV of dogs, and will focus on developing practical guidance for field implementation of ORV as a complementary measure to parenteral vaccinations. The revised recommendations will be published as a formal Tripartite publication in 2023, and this will be made available on the UAR Forum website.

### Cross-cutting opportunities for rabies control

This workstream aims to develop a framework and scientific foundation to support countries in integrating rabies control with other goals, including the World Health Organization Roadmap for Neglected Tropical Diseases 2021–2030 (44). While there have been multiple attempts to combine rabies control with other interventions (e.g., snakebite envenomation or parasitic diseases), there is a need to summarize the nature and findings of these approaches to understand their feasibility and added value. This workstream is conducting a scoping review of published and gray literature on cross-cutting approaches to rabies control to compile the evidence. The review will be complemented with a selection of key informant interviews and focus groups, facilitated by the UAR network, that look to capture unpublished experiences.

An additional component of this workstream will be building on the experiences of Sierra Leone and Liberia in conducting integrated rabies and Peste des petits ruminants (PPR) vaccination. Reports from Chief Veterinary Officers in these countries reported improved community participation and awareness resulting from joint campaigns, and noted that partnering with the public health sector had marked logistical and infrastructural benefits that enhanced cost-effectiveness and improved intersectoral collaboration (45). Through support from FAO and WOAH, this project will build on these successes in Liberia and Sierra Leone, and expand this to include Guinea, to support coordinated implementation of joint rabies and PPR vaccination programmes. The evidence generated from this project will help to inform the development of UAR Forum recommendations and potential future frameworks for where adoption of integrated approaches may be beneficial.

## Next steps

As the UAR Forum comes to the close of its second year, the Steering Group and Working Groups met during an operational meeting in December 2022 to review the governance, mode of operation and priority areas of the Forum. The aim of this meeting was to review the achievements of the Forum to date, and how to objectively measure outcomes from and maximize future value and impact of these outputs; identify challenges and barriers to progressing ongoing activities, reviewing if these proposed activities are still priorities, and if so, finding solutions in overcoming these barriers; identifying key priority activities for the UAR Forum in the coming 12 months based on reported country needs and developing a concrete work plan to address these; and finally restructuring the Forum to better meet the needs of the global community and support countries in reaching *Zero by 30*.

Key priorities will include focusing on engagement and empowerment of national stakeholders and local authorities, for example *via* the associations of mayors of large cities, while facilitating in-country support, particularly in the development of national strategic plans for rabies control. The UAR Forum will continue to promote the WOAH endorsement (9) of these plans and aim to link these with the agenda of Gavi, The Vaccine Alliance on PEP access (7). Gavi announced in 2019 that it would extend its portfolio to include human PEP, but noted that this would need to be part of an integrated One Health approach (46). While the roll out of this was delayed due to the COVID-19 pandemic, the inclusion of rabies PEP in this portfolio is critical for countries to implement effective One Health rabies control strategies, and the UAR Forum will continue to engage with Gavi to progress this.

In the long term the UAR Forum will continue to provide a platform to strengthen collaboration and coordination among partners, helping to reduce fragmentation, improve cross-sector efforts and facilitate country access to technical expertise. In doing so the UAR Forum will support countries to implement sustained rabies control measures and accelerate progress toward *Zero by 30*, while also building the One Health networks and capacity that are needed to improve the response to other endemic and emerging infectious diseases. In addition, having a central, coordinated network to implement rabies control will provide a more attractive and sustainable option for resource partners to invest in, helping mobilize resources to drive progress toward *Zero by 30*. The UAR Forum is supported by rabies experts that volunteer their time to support the *Zero by 30* agenda; while this approach ensures that any willing volunteer can participate, the sustainability and effectiveness of this approach must be closely monitored. Financial support for organizing the UAR Forum is critical, as key organization requirements such as staffing and website support must be supported to maintain the Forum.

## Conclusion

The UAR Forum is committed to actively supporting countries in their efforts to reach the goal of *Zero by 30*. By bringing together a diverse range of stakeholders and experts with a shared vision for the elimination of human deaths from dog-mediated rabies, the UAR Forum is putting One Health into practice and providing concrete outputs to support countries' rabies control efforts. A critical component of this will be ensuring that participants and organizations from canine rabies endemic countries are engaged, and actively participating in the UAR Forum. This will be a priority activity for the coming 12 months, with the UAR Forum exploring ways to better connect country needs with UAR Forum activities, in order to inform the development of outputs that can be most impactful for where they are needed most. In doing so, the UAR Forum will make a significant contribution to health equity and stronger human and animal health systems, and can provide a model for One Health implementation.

## Data availability statement

The original contributions presented in the study are included in the article/supplementary material, further inquiries can be directed to the corresponding author.

## Author contributions

RT and AF contributed equally to the writing of this manuscript. All authors reviewed and approved the manuscript.

## References

[1] Hampson K Coudeville L Lembo T Sambo M Kieffer A Attlan M. Estimating the global burden of endemic canine rabies. PLoS Negl Trop Dis. (2015) 9:e0003709. 10.1371/journal.pntd.0003709 25881058 PMC4400070

[2] World Health Organization. WHO Expert Consultation on Rabies: Third Report. (2018). Available online at: https://apps.who.int/iris/handle/10665/272364 (accessed May 21, 2022).

[3] World Health Organization, Food and Agriculture Organization of the United Nations, World Organisation for Animal Health, Global Alliance for Rabies Control. Zero by 30: The Global Strategic Plan to end Human Deaths from Dog-mediated Rabies by 2030. (2018). Available online at: https://www.unitedagainstrabies.org/publications/zero-by-30-the-global-strategic-plan-to-end-human-deaths-from-dog-mediated-rabies-by-2030/ (accessed May 21, 2022).

[4] Tidman R Thumbi SM Wallace R de Balogh K Iwar V Dieuzy-Labaye I. United against rabies forum: the One Health concept at work. Front Public Health. (2022) 10:854419. 10.3389/fpubh.2022.854419 35493394 PMC9043483

[5] Food and Agriculture Organization of the United Nations, World Organisation for Animal Health, World Health Organization. Terms of Reference of United Against Rabies Forum. (2020). Available online at: https://www.unitedagainstrabies.org/governance-policies/united-against-rabies-forum-terms-of-reference/ (accessed May 21, 2022).

[6] World Health Organization, Food and Agriculture Organization of the United Nations, World Organisation for Animal Health. United Against Rabies Forum – One Health in Action. (2020). Available online at: https://www.unitedagainstrabies.org/uar-zero-by-30-forum-12-01-2021-final/ (accessed May 21, 2022).

[7] World Health Organization, Food and Agriculture Organization of the United Nations, World Organisation for Animal Health. United Against Rabies Forum 2021 Review. (2021). Available online at: https://www.unitedagainstrabies.org/uar-zero-by-30-forum-12-01-2021-final/ (accessed May 21, 2022).

[8] United Against Rabies. (2022). Tackling Rabies and Dog Population Management: the Role of Local Authorities. Available online at: https://www.unitedagainstrabies.org/events-courses/tackling-rabies-and-dog-population-management/ (accessed December 5, 2022).

[9] World Organisation for Animal Health. Official Disease Status. (2022). Available online at: https://www.woah.org/en/what-we-do/animal-health-and-welfare/official-disease-status/ (accessed December 5, 2022).

[10] Food and Agriculture Organization of the United Nations, World Organisation for Animal Health, World Health Organization. Taking a Multisectoral, One Health Approach: A Tripartite Guide to Addressing Zoonotic Diseases in Countries. (2019). Available online at: https://www.woah.org/app/uploads/2021/03/en-tripartitezoonosesguide-webversion.pdf (accessed December 5, 2022).

[11] Global Alliance for Rabies Control. Stepwise Approach Towards Rabies Elimination (SARE). Available online at: https://rabiesalliance.org/tools/planning-tools/sare (accessed December 5, 2022).

[12] Partners for Rabies Prevention. Blueprint for Rabies Prevention Control. (2017). Available online at: https://caninerabiesblueprint.org/ (accessed December 5, 2022).

[13] National Centre for Animal Health Department of Livestock Ministry of Agriculture Forests Royal Government of Bhutan. National Rabies Prevention and Control Plan. (2017). Available online at: https://rr-asia.woah.org/wp-content/uploads/2020/03/rabies-plan_bhutan.pdf (accessed December 5, 2022).

[14] Department of Health Philippines and Department of Agriculture Bureau of Animal Industry. National Rabies Prevention and Control Program. (2019). Available online at: https://rr-asia.woah.org/wp-content/uploads/2020/03/final-mtp-rabies_philippines.pdf (accessed December 5, 2022).

[15] Centro Nacional de Programas Preventivos y Control de Enfermedades. Eliminación de la Rabia Humana Transmitida por Mordedura de Perro. (2018). Available online at: http://www.cenaprece.salud.gob.mx/descargas/pdf/PAE_PrevencionControlRabiaHumana2013_2018.pdf (accessed December 5, 2022).

[16] Ministério da Saúde. Normas Técnicas da Profilaxia da Raiva Humana. Available online at: https://www.gov.br/saude/pt-br/centrais-de-conteudo/publicacoes/publicacoes-svs/raiva/normas-tecnicas-da-profilaxia-da-raiva-humana.pdf/view (accessed December 5, 2022).

[17] World Health Organization. Toolkit to Develop a National Strategic Plan for TB Prevention, Care and Control. (2015). Available online at: https://www.who.int/publications/i/item/9789241507974 (accessed December 5, 2022).

[18] Uganda One Health Platform. Uganda One Health Strategic Plan 2018-2022. Available online at: https://www.health.go.ug/cause/uganda-one-health-strategic-plan-2018-2022/ (accessed December 5, 2022).

[19] United Against Rabies. A Generic Template for Developing a National Strategic Plan to Eliminate Rabies. (2021). Available online at: https://www.unitedagainstrabies.org/uar-best-practice/template-for-national-strategic-plan-to-control-rabies/ (accessed May 21, 2022).

[20] World Health Organization. WHO Recommended Strategies for the Prevention and Control of Communicable Diseases. (2001). Available online at: https://apps.who.int/iris/bitstream/handle/10665/67088/WHO_CDS_CPE_SMT_2001.13.pdf;jsessionid=BED34141F8084A2A236E381DC2FF3378?sequence=1 (accessed December 5, 2022).

[21] United States Council of State and Territorial Epidemiologists. Public Health Reporting and National Notification for Animal Rabies. (2010). Available online at: https://cdn.ymaws.com/www.cste.org/resource/resmgr/PS/09-ID-12.pdf (accessed December 5, 2022).

[22] World Organisation for Animal Health. Terrestrial Animal Health Code. (2022). Available online at: https://www.woah.org/en/what-we-do/standards/codes-and-manuals/terrestrial-code-online-access/ (accessed December 5, 2022).

[23] National Association of State Public Health Veterinarians. Compendium of Animal Rabies Prevention and Control. (2016). Available online at: https://ldh.la.gov/assets/oph/Center-PHCH/Center-CH/infectious-epi/VetInfo/Rabies/RabiesCompendium2016.pdf (accessed December 5, 2022).

[24] World Organisation for Animal Health. World Animal Health Information System. (2022). Available online at: https://www.woah.org/en/what-we-do/animal-health-and-welfare/disease-data-collection/world-animal-health-information-system/ (accessed December 5, 2022).

[25] World Health Organization. Global Health Observatory Data Repository. (2022). Available: https://apps.who.int/gho/data/node.main.NTDRABIESHUMANDEATHS?lang=en (accessed December 5, 2022).

[26] Friedrich-Loeffler-Institut. Rabies Bulletin Europe. (2022). Available online at: https://rbe.fli.de/ (accessed December 5, 2022).

[27] Centers for Disease Control and Prevention. Nationa Notifiable Diseases Surveillance System (NNDSS). (2022). Available online at: https://www.cdc.gov/nndss/index.html (accessed December 5, 2022).

[28] Pan American Health Organization. SIRVERA. (2022). Available online at: https://www.paho.org/en/topics/rabies (accessed December 5, 2022).

[29] Global Alliance for Rabies Control. A Rabies Epidemiological Database for sub-Saharan Africa. Available online at: https://rabiesalliance.org/networks/paracon/bulletin (accessed December 5, 2022).

[30] United Against Rabies. Minimum Data Elements. (2022). Available online at: https://www.unitedagainstrabies.org/uar-best-practice/minimum-data-elements (accessed 1 August 2022).

[31] United Against Rabies. Toolbox. (2022). Available online at: https://www.unitedagainstrabies.org/resources-toolbox/ (accessed May 21, 2022).

[32] United Against Rabies. The Rabies Roadmap. (2022). Available online at: https://www.unitedagainstrabies.org/the-rabies-roadmap/ (accessed May 21, 2022).

[33] Food and Agriculture Organization of the United Nations, United Nations Environment Programme, World Organisation for Animal Health, World Health Organization. One Health Joint Plan of Action (2022-2026): Working Together for the Health of Humans, Animals, Plants and the Environment. (2022). Available online at: https://www.who.int/publications/i/item/9789240059139 (accessed December 5, 2022).

[34] United Against Rabies. Template for Landscaping of Resource Partners for Rabies. (2022). Available online at: https://www.unitedagainstrabies.org/uar-best-practice/landscaping-of-resource-partners-for-rabies-template/ (accessed December 5, 2022).

[35] United Against Rabies. News and Case Studies. (2022). Available online at: https://www.unitedagainstrabies.org/news-and-case-studies/ (accessed November 10, 2022).

[36] United Against Rabies. Pitch Deck. (2022). Available online at: https://www.unitedagainstrabies.org/uar-best-practice/pitch-deck/ (accessed December 5, 2022).

[37] Riccò M Ranzieri S Peruzzi S Valente M Marchesi F Bragazzi NL. Antigen detection tests for SARS-CoV-2: a systematic review and meta-analysis on real world data. Acta Biomed. (2022) 93:e2022036. 10.23750/abm.v93i2.11031 35546034 PMC9171867

[38] Yilma T Aziz F Jones L Jones L Ngotho R Wamwayi H. Inexpensive vaccines and rapid diagnostic kits tailor-made for the global eradication of rinderpest, and technology transfer to Africa and Asia. Dev Biol. (2003) 114:99–111. 14677681

[39] Tenzin T Lhamo K Rai P Tshering D Jamtsho P Namgyal J. Evaluation of a rapid immunochromatographic test kit to the gold standard fluorescent antibody test for diagnosis of rabies in animals in Bhutan. BC Vet Res. (2020) 8:183. 10.1186/s12917-020-02405-4 32513172 PMC7281917

[40] Yale G Gibson A Mani R Harsha PK Costa NC Corfmat J. Evaluation of an immunochromatographic assay as a canine rabies surveillance tool in Goa, India. Viruses. (2019) 11:649. 10.3390/v11070649 31311178 PMC6669590

[41] Kimitsuki K Saito N Yamada K Park CH Inoue S Suzuki M. Evaluation of the diagnostic accuracy of lateral flow devices as a tool to diagnose rabies in post-mortem animals. PLoS Negl Trop Dis. (2020) 14:e0008844. 10.1371/journal.pntd.0008844 33151941 PMC7671516

[42] Mananggit M Manalo D Saito N Kimitsuki K Garcia AMG Lacanilao PMT. Lateral flow devices for samples collected by straw sampling method for postmortem canine rabies diagnosis. PLoS Negl Trop Dis. (2021) 15:e0009891. 10.1371/journal.pntd.0009891 34882672 PMC8659307

[43] World Health Organization. Oral Vaccination of Dogs Against Rabies. (2007). Available online at: https://apps.who.int/iris/bitstream/handle/10665/331036/WHO-HTM-NTD-NZD-2007.1-eng.pdf (accessed December 5, 2022).

[44] World Health Organization. Ending the neglect to attain the Sustainable Development Goals: A Road Map for Neglected Tropical Diseases 2021-2030. (2021). Available online at: https://www.who.int/publications/i/item/9789240010352 (accessed May 21, 2022).

[45] Food and Agriculture Organization of the United Nations. Panel Discussion on PPR and Rabies Vaccination Highlights Diverse One Health Approaches to Health Service Delivery. (2022). Available online at: https://www.fao.org/ppr/news-and-events/news/detail/en/c/1469646/ (accessed December 5, 2022).

[46] Food and Agriculture Organization of the United Nations, World Organisation for Animal Health, World Health Organization, Global Alliance for Rabies Control. United Against Rabies Collaboration First Annual Progress Report. (2019). Available online at: https://www.unitedagainstrabies.org/who-cds-ntd-nzd-2019-04-eng/ (accessed May 21, 2022).

